# Birth defects in HIV vertically exposed children

**DOI:** 10.1186/1471-2334-14-S4-O7

**Published:** 2014-05-29

**Authors:** Ana Maria Tudor, Mariana Mărdărescu, Ioana Alina Anca, Cristina Petre, Cosmina Cristea, Ruxandra Neagu-Drăghicenoiu, Rodica Ungurianu

**Affiliations:** 1 Carol Davila University of Medicine and Pharmacy, Bucharest, Romania; 2 National Institute for Infectious Diseases “Prof. Dr. Matei Balş”, Bucharest, Romania; 3 Institute for Mother and Child Protection “Alfred Rusescu”, Bucharest, Romania

## 

Objective: to identify types of birth defects in HIV vertically exposed children and to determine the rate of congenital disorders counted in children born to HIV infected mothers.

We analyzed the data recorded for HIV perinatally exposed children followed up in the National Institute for Infectious Diseases “Prof. Dr. Matei Balş”, Bucharest, the Pediatric Department from January 1^st^ 2006 to December 31^st^ 2012.

Of 203 children with data on clinical, imagistic and virologic aspects, 20% were diagnosed with HIV infection and more than 33% had at least one congenital condition.

The birth defects identified in studied children were: congenital heart defects 63%, musculoskeletal defects 23%, renal malformations 10%, neurologic defects 10%, digestive tract malformations 5%, metabolic and storage disorders 2% and genetic disorders 2%. 9% from studied children with birth defects had more than one organ involvement.

Among HIV infected children the most frequent congenital disease was heart malformation, followed by renal and neurologic malformations. In total less than 30% from HIV vertical infected children and more than 33% HIV exposed but negative children had congenital diseases. The difference is not statistically significant (p=0.38). Comparing the group of children born by treated mothers to those from untreated mothers we have noticed that the use of antiretrovirals during or before pregnancy is not associated with malformations (p=0.45). In the same time the risk of congenital diseases in our patients is associated with mothers being part of Romanian cohort (p=0.05). The relative risk to have malformations in the second generation of Romanian cohort is 1.49 higher comparing with children born by more recently infected mothers. We also found very rare congenital diseases: chromosomal hermaphroditism, gangliosidosis and Niemann Pick syndrome (less than 1/100,000 live births).

The rate of malformations is relatively high among HIV exposed children compared with general population in Romania and it was associated with long history of HIV disease (Romanian cohort).

